# Tonsillectomy versus Tonsillotomy for Sleep-Disordered Breathing in Children: A Meta Analysis

**DOI:** 10.1371/journal.pone.0121500

**Published:** 2015-03-25

**Authors:** Hui Wang, Yangyang Fu, Yanmei Feng, Jian Guan, Shankai Yin

**Affiliations:** 1 Department of Otolaryngology, Shanghai Jiao Tong University Affiliated Sixth People’s Hospital, Shanghai, China, 200233; 2 Department of Otolaryngology, Dalian Municipal Central Hospital, 826 Xinan Road, Dalian, 116033 China; Central South University, CHINA

## Abstract

**Objectives:**

Tonsillotomy has gained popular acceptance as an alternative to the traditional tonsillectomy in the management of sleep-disordered breathing in children. Many studies have evaluated the outcomes of the two techniques, but uncertainty remains with regard to the efficacy and complications of tonsillotomy versus a traditional tonsillectomy. This study was designed to investigate the efficacy and complications of tonsillotomy versus tonsillectomy, in terms of the short- and long-term results.

**Methods:**

We collected data from electronic databases including MEDLINE, EMBASE, and the Cochrane Library. The following inclusion criteria were applied: English language, children, and prospective studies that directly compared tonsillotomy and tonsillectomy in the management of sleep disordered breathing. Subgroup analysis was then performed.

**Results:**

In total, 10 eligible studies with 1029 participants were included. Tonsillotomy was shown to be advantageous over tonsillectomy in short-term measures, such as a lower hemorrhage rate, shorter operation time, and faster pain relief. In long-term follow-up, there was no significant difference in resolution of upper-airway obstructive symptoms, the quality of life, or postoperative immune function between the tonsillotomy and tonsillectomy groups. The risk ratio of SDB recurrence was 3.33 (95% confidence interval = 1.62 6.82, P = 0.001), favoring tonsillectomy at an average follow-up of 31 months.

**Conclusions:**

Tonsillotomy may be advantageous over tonsillectomy in the short term measures and there are no significant difference of resolving obstructive symptoms, quality of life and postoperative immune function. For the long run, the dominance of tonsillotomy may be less than tonsillectomy with regard to the rate of sleep-disordered breathing recurrence.

## Introduction

Most tonsillectomies are performed in children with sleep-disordered breathing (SDB) to reduce obstruction due to hypertrophic tonsils. The main drawbacks of tonsillectomy are the potential for serious postoperative bleeding, with an estimated frequency of 1–20% [1, 2], postoperative pain, eating and drinking difficulties, and reduced immune function in the early stages after the operation[3].

Tonsillotomy (partial tonsillectomy, intracapsular tonsillectomy, subtotal tonsillectomy) became popular in the late 1980s because it caused less pain, had an easier recovery, and allowed greater retention of immune function [4, 5]. In recent years, children with SDB have benefited from the less invasive tonsillotomy [6]. Tonsillotomy patients experience less pain, equivalent or easier recovery, better food intake, and maintain the immunological function of the tonsils, while being as effective as tonsillectomy for resolving upper-airway obstructive symptoms for SDB in children.

Many studies have evaluated the outcomes of the two techniques, but uncertainty remains with regard to the efficacy and complications of tonsillotomy versus a ‘traditional’ tonsillectomy. The “gold standard” for measuring SDB is overnight polysomnography (PSG). However, the actual rate of ‘cure,’ measured by PSG changes in children, remains controversial [7]. Children with tonsillar hypertrophy and SDB show the impact of health-related quality of life before surgery and improve dramatically after both tonsillotomy and tonsillectomy. Some studies have reported the recurrence of obstructive symptoms due to regrowth of the remaining tonsillar tissue and recurrent tonsillitis over a longer period after tonsillotomy [8]. Thus, it is important to evaluate tonsillotomy and tonsillectomy comprehensively, especially paying attention to short-term and long-term results.

The objective of this study was to systematically review the two tonsil surgery techniques, considering the short-term effects, including secondary postoperative bleeding, pain-free days, and operation time, and to perform a subgroup analysis of resolution of upper-airway obstructive symptoms, including PSG outcomes, quality of life (QoL), immune function, and the rate of sleep-disordered breathing (SDB) recurrence following tonsillotomy versus tonsillectomy over the longer term.

## Methods

The objective of this systematic review with a meta-analysis was to analyze the currently available data and to compare the effects in terms of resolving obstructive symptoms with tonsillotomy, compared with traditional tonsillectomy, in children, with regard to the short- and long-term outcomes. We conducted key words searches in the biomedical electronic databases, including MEDLINE, EMBASE, and the Cochrane Library: (partial tonsillectomy or tonsillotomy or partial intracapsular tonsillectomy or subtotal tonsillectomy) and (tonsillectomy or total tonsillectomy) in combination with (obstructive sleep apnea hypopnea syndrome or OSAHS or obstructive sleep apnea syndrome or OSAS or obstructive sleep apnea or OSA or sleep apnea or apnea or sleep disorders breathing or SDB) were used as research terms.

### Inclusion and Exclusion Criteria

The following inclusion criteria were used: English language, children (birth to 18 years), and concerning complications and effects of tonsillotomy and tonsillectomy in the management of SDB. The specific search terms used were intracapsular tonsillectomy, partial tonsillectomy, tonsillotomy, tonsillectomy and the children with obstructive sleep apnea or sleep disorders breathing. Studies that compared tonsillotomy and tonsillectomy directly were included. Those that described only tonsillotomy or tonsillectomy were excluded. Only the prospective studies were included. Manual checks of the bibliographies of all articles were performed. We found no internal control patient studies (studies in which one tonsil was removed via tonsillotomy and the other via tonsillectomy). The final search was updated in June 2014. The results of these studies were combined in an evidence table and a subgroup analysis was performed with secondary postoperative bleeding, pain-free days, operation time, resolution of upper-airway obstructive symptoms, including PSG outcomes, QoL, and immune function, and the rate of SDB recurrence. All studies involved a minimum of 10 patients.

### Data Extraction and Quality Assessment

Two investigators (Dr. Wang and Fu) performed the data collecting, data screening, and data extraction. Any disagreement was settled by a discussion with a third reviewer (Dr Guan) The following data were extracted from the original studies: first author, year of publication, country, sample size, age, gender, surgery allocation, study design, follow up, surgery technique, the percentage of secondary postoperative bleeding, pain-free days, immune function(including IgA, IgG, IgM), apnea-hypopnea index (AHI), operation time, the percentage of SDB recurrence, QoL measured by the GCBI scale. We performed the quality assessment of the included study with the Newcastle-Ottawa Scale(www.ohri.ca/programs/clinical_epidemiology/oxford.htm). The Newcastle-Ottawa Scale rated the quality according to the following items: selection, study comparability, follow-up, and outcome of interest. The scale≥6 were considered to indicate ‘high’ quality. Quality assessment was independently evaluated by two reviewers (Dr. Wang and Fu).

The Review Manager software (ver. 5.2; Cochrane Collaboration, Oxford, UK) was used for statistical analyses. The Mantel-Haenszel fixed-effects model or the random-effects model was used to calculate summary effect measures (risk ratio and risk difference) with corresponding 95% confidence intervals (CI), and forest plots were generated. Homogeneity was tested using Cochrane χ^2^ tests. Risk ratios and risk differences were generated comparing tonsillotomy to tonsillectomy. A p value of < 0.05 was considered to indicate statistical significance.

## Results

### Search Results

In total, 199 studies were initially identified by electronic and manual searching. Those reporting the outcome measures of operation time, secondary postoperative bleeding, pain-free days, PSG outcomes, QoL, immune function, and the rate of SDB recurrence were included. The duration of follow-up was 10 days to 72 months. At the first stage of screening and according to the inclusion criteria, 93 studies were excluded for the following reasons: not English language, reviews, letters, case reports, and repeated references. At the second stage of screening and by the exclusion criteria, after reading the abstracts or full articles, 96 studies were excluded for the following reasons: non-prospective case series, those that described tonsillotomy or tonsillectomy only, lacked complete data, concerned suspected tonsillar tumor, and patients with cleft palate or skeletal dysplasia. The scale evaluating the quality of life in one study differed from the others, so that study was excluded [9].

**Fig 1:**
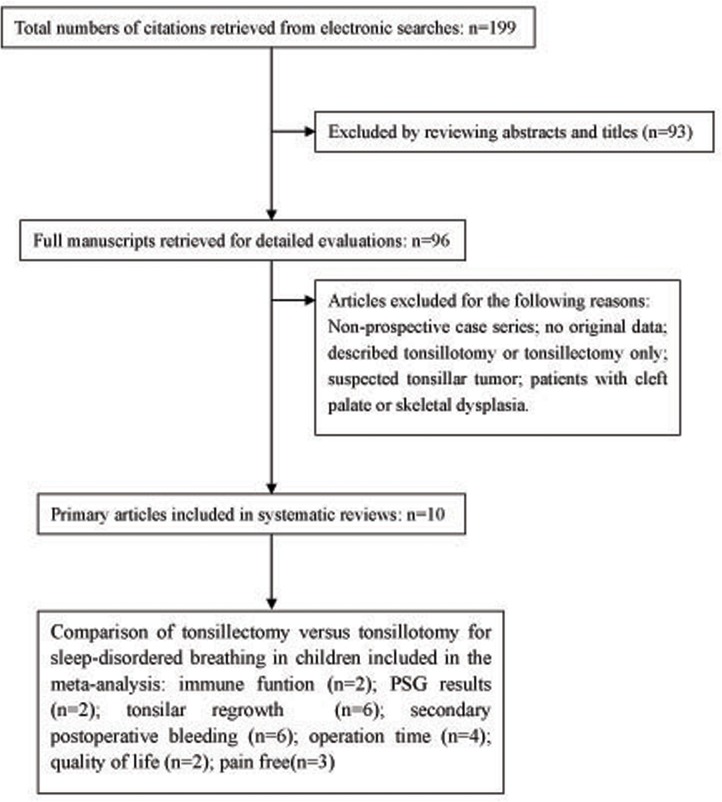
Search methodology.

Finally, 10 studies met the inclusion criteria and were included in our meta-analysis (Table 1). Of the 10 studies, 6 included data for secondary postoperative bleeding[6, 10–14], 6 data for SDB recurrence[6, 11–15], 3 data for pain-free days[11, 12, 16], 2 data for immune function[6, 17], 4 data for operation time[6, 10, 12, 13], 2 data for PSG outcomes[6, 10], and 2 data for QoL[11, 18] (Fig. 1). The details were displayed in Table 2.

**Table 1:** Evidence table showing the characteristics of the included studies.

Study	Technique		Sample Size		Location	Age		Allocation	Data Collection	Scoring	Follow-up
	TT	TE	TT	TE		TT	TE				
Hultcrantz1999[13]	CO2 laser	CO2 laser	21	20	Sweden	6±1.5	6±1.5	randomized	prospective	7	12m
Hultcrantz 2005[17]	CO2 laser	CO2 laser	21	20	Sweden	3–9	3–9	randomized	prospective	7	72m
Ericsson 2006[19]	Surgitrone	cold knife blunt dissection	49	43	Sweden	8.7±3.6	9.8±3.4	randomized	prospective	8	10days
Rwichel 2007[15]	CO2 laser	blunt dissection	49	64	Germany	4.5	4.9	non randomized	prospective	9	24m
Ericsson 2009[12]	Surgitrone	cold knife blunt dissection	35	32	Sweden	4.5–5.5	4.5–5.5	randomized	prospective	8	6m
Wood 2011[18]	Coblation	Coblation	63	118	Australia	5	5.7	non randomized	prospective	7	24m
Cantarella 2012[11]	Gyrus microdebrider	cold knife blunt dissection	14	15	Italy	5.1±1.7	5.2±1.8	non randomized	prospective	7	6m
Moriniere 2013[14]	Ring electrode	bipolar scissors	88	105	France	4.88 ±2.6	4.75±2.37	non randomized	prospective	8	12m
Dai 2014 [16]	Coblation	Coblation	37	20	China	5	4.6	randomized	prospective	7	3m
Zhang 2014 [6]	Coblation	Coblation	82	133	China	4.8	6.4	nonrandomized	prospective	8	64.3m

**Table 2:** The study included the outcome measures in subgroup analysis.

Study	secondary postoperative bleeding	SDB recurrence	pain-free days	immune function	operation times	PSG outcomes	quality of life
Hultcrantz1999[13]	Yes	Yes	Yes		Yes		
Hultcrantz 2005[17]		Yes					
Ericsson 2006[19]			Yes				
Rwichel 2007[15]	Yes	Yes					
Ericsson 2009[12]	Yes	Yes	Yes				Yes
Wood 2011[18]							Yes
Cantarella 2012[11]	Yes				Yes	Yes	
Moriniere 2013[14]	Yes	Yes			Yes		
Dai 2014 [16]				Yes			
Zhang 2014 [6]	Yes	Yes		Yes	Yes	Yes	

### Study Characteristics

All 10 eligible studies were published between 1999 and 2014. Two studies were from China[6, 17], four from Sweden[11, 12, 15, 16], and one each from Italy[10], France[13], Australia[18], and Germany[14]. The mean number of subjects in each study was 46.5 (range, 14–133), with the mean number of tonsillotomy patients being 45.9 (range, 14–82) and tonsillectomy patients being 47 (range, 15–133). The mean age of all patients was 5.37 (range, 2–15) years, with tonsillotomy patients averaging 4.95 years and tonsillectomy patients averaging 5.79 years. The average length of follow-up was 22.18 months (10 days to 72 months). All 10 studies were prospective; among them, five were randomly allocated, and five were non-randomized. All 10 prospective studies reported losses to follow-up of < 10%.

### Fixed-Effects Modeling of Primary Outcome Measures

Fixed-effects modeling was performed to estimate summary effect measures, comparing tonsillotomy to tonsillectomy for the outcomes of secondary postoperative bleeding rate, and effects in resolving upper-airway obstructive symptoms, including PSG outcome, immune function (including IgA, IgG, and IgM), and rate of SDB recurrence in long-term follow-up. Regarding the procedure time and QoL (measured using the Glasgow Children’s Benefit Inventory (GCBI) scale after the two procedures) data, the I^2^ measure of heterogeneity was >50%, suggesting strong heterogeneity among the studies. Thus, the data were analyzed using a random-effects model.

**Fig 2:**
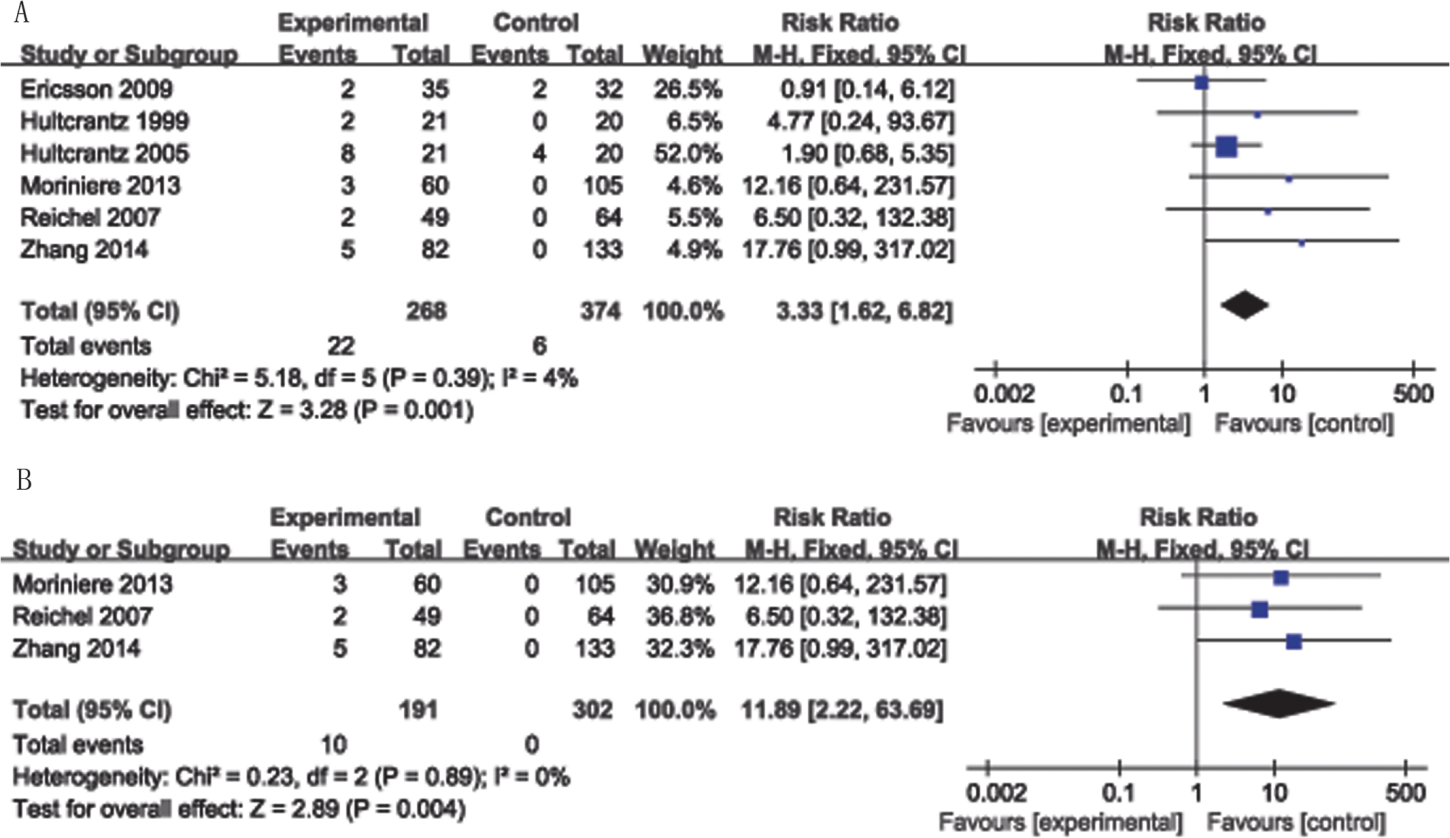
Forest plot of included studies demonstrated the risk ratio for the rate of SDB recurrence for tonsillotomy versus tonsillectomy. A risk ratio > 1 favors tonsillectomy. A: All randomized and non-randomized studies of SDB recurrence were included. B: Only non-randomized studies of SDB recurrence were included.

**Fig 3:**
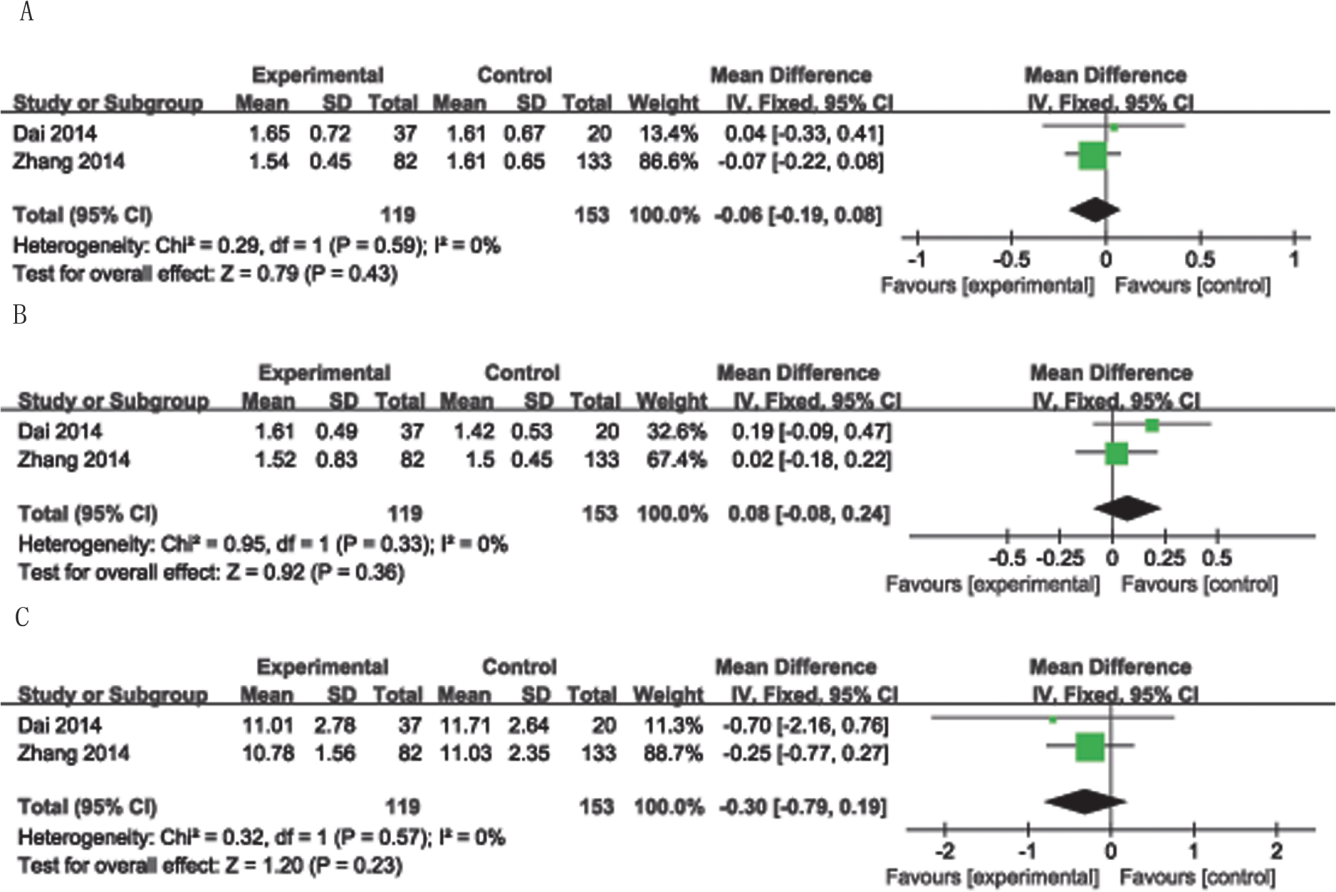
Forest plot of included studies demonstrated the risk ratio for immune function for tonsillotomy versus tonsillectomy. A: IgA, B: IgM, C: IgG.

**Fig 4:**
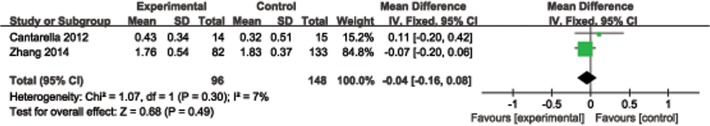
Forest plot of included studies demonstrated the risk ratio for PSG results for tonsillotomy versus tonsillectomy.

The risk ratio, as an estimate of summary effect measures, was calculated from the included studies directly comparing the two tonsil removal techniques. The risk ratio of secondary postoperative bleeding of tonsillotomy versus tonsillectomy was 0.28 (95% CI = 0.1–0.78, P = 0.01, I^2^ = 0%, p < 0.001), significantly favoring tonsillotomy. There were 268 children in the tonsillotomy group, 22 children developed the recurrence of sleep disorder breathing. The rate of recurrence is 8.2% (22/268); There were 374 children in the tonsillectomy group, 6 children developed the recurrence of sleep disorder breathing. The rate of recurrence is 1.6% (6/374). The risk ratio for the rate of SDB recurrence was 3.33 (95% CI = 1.62–6.82, P = 0.001, I^2^ = 4%), significantly favoring TE (Fig. 2A). There were three non-randomized trials. In those studies, children with obstructive problems (snoring, sleep apnea, mouth breathing) due to tonsillar hyperplasia (none had a history of repeated throat infections) were treated by tonsillotomy. The children with recurrent tonsillitis underwent TEs. In a non-randomized trial subgroup analysis, the risk ratio for the rate of SDB recurrence of tonsillotomy versus tonsillectomy was 12.56 (95% CI = 2.28–69.15, P = 0.004, I^2^ = 0%; Fig. 2B), also significantly favoring tonsillectomy. There appeared to be no clinically significant difference in immune function (IgA, RR = −0.06, 95% CI = −0.19 to 0.08, P = 0.43, I^2^ = 0%, Fig. 3B; IgG, RR = −0.08, 95% CI = −0.08 to 0.24, P = 0.36, I^2^ = 0%, Fig. 3A; IgM, RR = −0.3, 95% CI = −0.79 to 0.19, P = 0.23, I^2^ = 0%, Fig. 3C). There was also no significant difference in PSG outcomes (RR = −0.04, 95% CI = −0.16 to 0.08, P = 0.49, I^2^ = 7%, Fig. 4). The risk ratio for QoL, measured by the GCBI scale, after the two procedures was −0.97 (95% CI = −18.63 to 16.70, P = 0.91, I^2^ = 99%); there was no significant difference between tonsillotomy and tonsillectomy.

**Fig 5:**
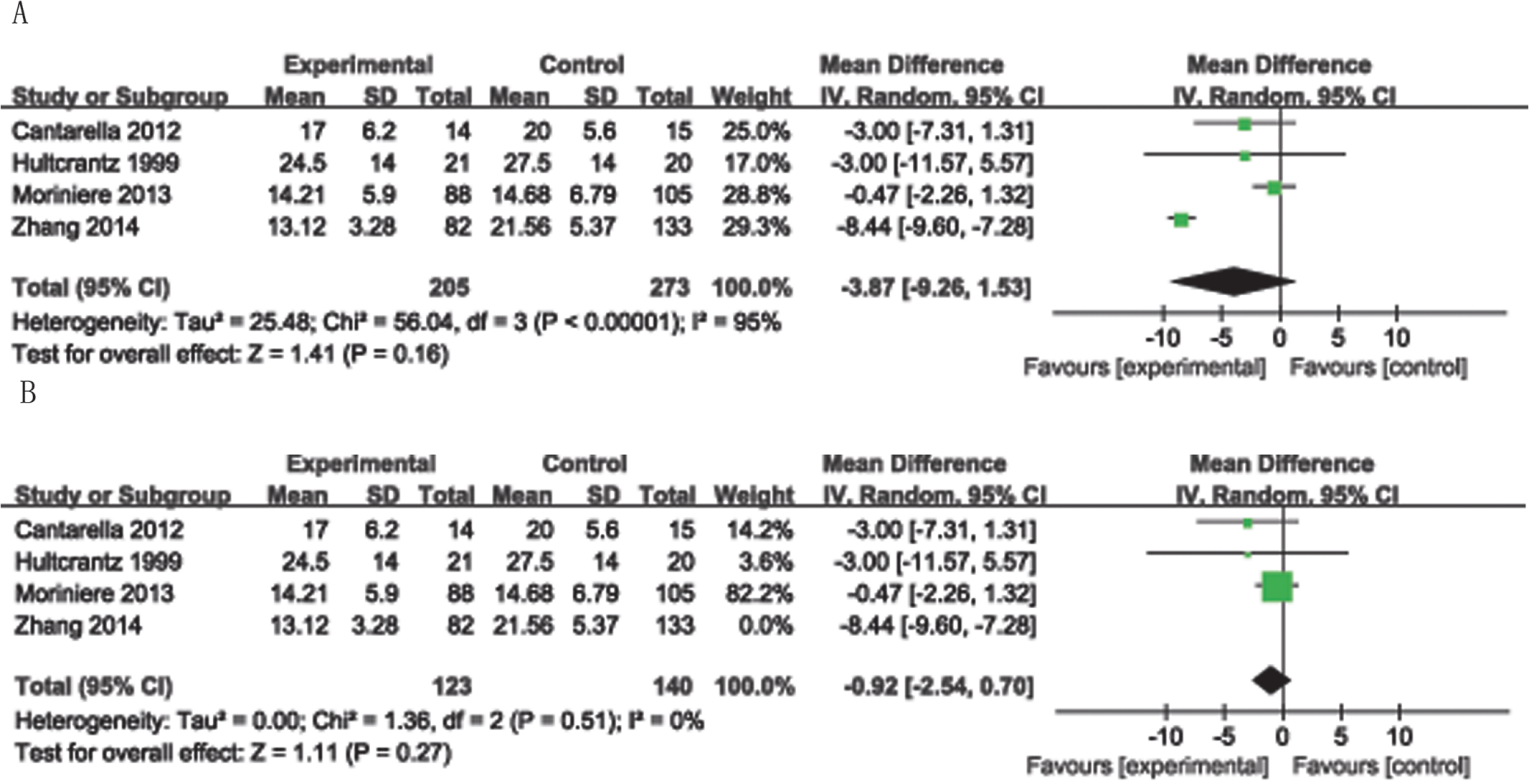
Forest plot of included studies demonstrated the risk ratio for operation time for tonsillotomy versus tonsillectomy. A: All studies were included. B: Studies, with the exception of the Coblation technique, of tonsillotomy versus tonsillectomy.

There was no significant difference in procedure time (RR = −3.87, 95% CI = −9.26 to 1.53, P = 0.16, I^2^ = 95%; Fig. 5A). Significant heterogeneity was seen in procedure time, mostly due to the differing surgical methods. Likewise, there was a significant difference in the number of pain-free days after the procedure (RR = −1.66, 95% CI = −3.23 to −0.1, P = 0.004, I^2^ = 0%).

### Heterogeneity and Quality Assessment Results

We used the I^2^ index to explore variability in effect-size estimates among studies. The index values for the differences between tests and controls in the subgroups of secondary postoperative bleeding rates, PSG outcomes, immune function, including IgA, IgG, IgM, pain-free days, and the rate of SDB recurrence were 0%, 7%, 0%, 0%, 0%, 0%, and 4%, respectively, indicating that the studies were homogeneous in nature. The shapes of the funnel plots revealed no obvious asymmetry. However, there was significant heterogeneity in procedure time (95%). We excluded data from a report that used the Coblation technique in tests and controls [6], and the I^2^ index changed to 0% (Fig. 5B). The Coblation procedure is minimally invasive and causes low-temperature molecular disintegration with minimal necrosis of the surrounding tissue. The average procedure time was shorter than with a traditional tonsillectomy. Also, significant heterogeneity in QoL, measured by the GCBI scale, was seen after the two techniques, possibly due mainly to the duration of follow-up. The Newcastle-Ottawa Scale rated the quality of the included study was shown in Table 1. The included studies were high quality, as evidenced by the Newcastle-Ottawa Scale scores ranged from 7to 9,with a median of 7.6.

## Discussion

The present study demonstrated that tonsillotomy might be advantageous over tonsillectomy in the following short-term measures: a lower hemorrhage rate, shorter procedure time, and more rapid recovery to a pain-free state. At long-term follow-up, there was no significant difference in terms of resolution of upper-airway obstructive symptoms, the quality of life, or postoperative immune function, but a difference with regard to the rate of sleep-disordered breathing recurrence between the tonsillotomy and tonsillectomy groups in the subgroup analysis. Tonsillotomy is as effective as tonsillectomy for the long-term management of children suffering from SDB due to hypertrophic tonsils, as evidenced by the PSG and QoL results. In the present study, we found no significant difference in resolution of SDB symptoms between tonsillotomy and tonsillectomy after follow-up of 3 months to more than 60 months. All included studies reported relief of obstructed breathing at 7–10 days after the surgery in both groups. There was no significant difference between the two techniques, as evidenced by PSG measurements. Regarding health-related QoL, removing only the protruding parts of the tonsils with tonsillotomy seems to have the same benefit in terms of QoL as a regular tonsillectomy. In the prospective studies, parental reporting of changes in QoL using the GCBI was used to compare tonsillotomy and tonsillectomy procedures for the treatment of SDB. The present meta-analysis demonstrated that in children with SDB, the improvements in all subscores of the GCBI indicated significant health benefits of both tonsillotomy and tonsillectomy; there was no significant difference between tonsillotomy and tonsillectomy with regard to overall improvement in QoL. This is consistent with comparative studies that used the Child Behavior Checklist (CBCL) [19–21].

Tonsillectomy is a common method for the treatment of SDB in children. However, opinions differ on its possible effects on immune function. Some believe that tonsillectomy impairs the development of the immune system in childhood in the early period and reaches ‘normal’ later [22]. The IgG, IgA, and IgM levels in patients undergoing tonsillectomy were lower 1 month after surgery compared with those in age-matched healthy controls [3]. In our previous study, in which serum IgG, IgA, IgM, C3, and C4 concentrations were measured, there was no significant difference between prior to and 1 month after both tonsillotomy and tonsillectomy [6]. In the present study, immunological function, as indicated by IgA, IgG, and IgM levels, was maintained at 3 months after the procedure in the children with both tonsillotomy and tonsillectomy, while the airway obstruction was relieved. There was no significant difference in immunological function between the children who underwent tonsillotomy and tonsillectomy, consistent with the conclusion reached by Böck [23].

At short term measures, there are various post-operative advantages to tonsillotomy: less pain, a lower hemorrhage rate and shorter procedure time. In tonsillotomy procedure, the exposure of the pharyngeal muscles is avoided, which maybe contribute to the rapid recovery and less pain. Moreover, no important blood vessels are damaged, which leads to the findings of less post-operative bleeding, as compared with tonsillectomy. At long-term follow-up, the recurrence of obstructive symptoms, indicated by snoring again, might occur after tonsillotomy due to tonsillar regrowth. The recurrence of symptoms was in accordance with data (4.1–38%) in the included reports from 6 months to 6 years after the tonsillotomy operation[14, 15]. The tonsil regrowth frequency and recurrence of symptoms was in accordance with data (0–4.7%) from previous reports [24]. In our study, the risk ratio of SDB recurrence was 3.33 between tonsillectomy and tonsillotomy, significantly favoring tonsillectomy. It remains unclear why palatine tonsils grow back in a small percentage of children. Many risk factors affect tonsillar regrowth, including age at the time of surgery, upper respiratory tract infections, and a history of allergies [25]. Younger children with tonsillary tissue that has not reached maximum size have an increased risk of regrowth. In some patients, chronic tonsillitis may not have been recognized prior to surgery and the remaining tonsillar tissue might be stimulated to become hyperplastic. This suggests that tonsillectomy should be performed following a diagnosis of SDB due to tonsillar hyperplasia when combined with recurrent tonsillitis and a history of allergies.

This study had several limitations. The number of pooled subjects was relatively small. Also, studies with negative results may not have been reported; this represents a risk of publication bias. Tonsillotomy with coblation technique has gained acceptance due to faster procedure time, which contribute to the significant heterogeneity in the analysis of operation time. As for the coblation technique, electrodes at the coblation probe tip emit radiofrequency energy at the target tissue. With the low temperature generated by coblation, small blood vessels are sealed during the process, which minimizes hemorrhage and minimized the operation time. Despite these limitations, our meta-analysis provided a reasonable method of evaluating the available data regarding tonsillotomy for SDB in children.

## Conclusions

In summary, tonsillotomy may be advantageous over tonsillectomy in terms of the short-term measures of lower hemorrhage rate, shorter procedure time, and reduced pain. There was no significant difference in terms of resolution of obstructive symptoms, quality of life, or postoperative immune function. For the long run, the dominance of tonsillotomy may be less than tonsillectomy with regard to the rate of sleep-disordered breathing recurrence.

## Supporting Information

S1 PRISMA Checklist Checklist for this meta-analysis. (DOC)

S1 Data Data for this meta-analysis. (XLS)
